# The Evolutionary and Developmental Pathways of Obsessive Compulsive Rituals in Psychopathology

**DOI:** 10.3390/brainsci14111118

**Published:** 2024-11-02

**Authors:** Matteo Tonna

**Affiliations:** 1 Psychiatric Unit, Department of Medicine and Surgery, University of Parma, 43126 Parma, Italy; matteo.tonna@unipr.it; 2 Department of Mental Health, Local Health Service, 43125 Parma, Italy

In this Special Issue, we promote a strictly dimensional approach to shed light on the clinical and developmental significance of Obsessive Compulsive Symptoms (OCS) across psychopathology. OCS, in fact, far from being limited to a diagnosis of Obsessive Compulsive Disorder (OCD), are spread across a wide range of neuropsychiatric conditions, from developmental years to adulthood. In childhood, OCD compulsions are continuous with the normative compulsive-like behaviors of early infancy [1]. After the age of 7, they often co-occur with different neurodevelopmental disorders, including Autism Spectrum Disorder (ASD) [2]. In this respect, Aymerich and co-workers (contribution 1) review the literature on the comorbidity between childhood OCD and ASD and their clinical and therapeutic implications. Longitudinally, childhood OCD increases the risk of a later onset of adult major endogenous psychoses [3], with a possible mediating role of trauma exposure [4]. In this vein, Borrelli and colleagues (contribution 2) elucidate the developmental trajectories linking OCD, childhood trauma experiences, and psychotic vulnerability.

Many of these neurodevelopmental disorders, which see OCD compulsions as behavioral output, insist on a disrupted multisensory grounding [5]. In humans, in fact, multisensory integration networks are more vulnerable to early deviations of normative developmental trajectories since their less constrained neural configurations are due to extensive neotenic synaptic plasticity [6]. Consistently, there is evidence for a strong association between OCS, in particular compulsive rituals, and the trait-like conditions of sensorial unbalance, for example, in the form of sensory over-responsivity [7] and impaired access to somatosensory states [8]. In this regard, Tal and colleagues (contribution 3) present their findings on the relationship between childhood OCD and atypical sensory processing.

These findings fit well with recent evolutionary models of compulsive rituals [9]. OCD compulsions present in fact a fixed motor pattern, which is homologous to that of ritual behavior across vertebrate phylogeny [10]. This specific, pre-programmed motor structure, packaged in basal ganglia loops, conserves the adaptive mechanism to cope with any unpredictable or “high-entropy” state, so to calibrate any behavior-environment mismatch and regain a feeling of order and control [11,12].

In psychopathology, OCD compulsions may therefore represent a preferential, highly conserved behavioral output [13], triggered either by sensory unpredictability due to different developmental pathways (biological or psychosocial), or by abrupt environmental unpredictability. This is the case, for example, of the exacerbation and clinical modification of OCD compulsions during the COVID-19 pandemic, as demonstrated by D’Urso and colleagues (contribution 4). Alternatively, compulsive rituals may be inappropriately or excessively released by primary dysregulation of cortico-striato-thalamocortical circuitry (CSTC), as in the case of “pure” OCD dispositions, or by any pathogenic mechanism or injury, eventually converging towards a fronto-striatal imbalance [14]. Grassi and Pampaloni (contribution 5), for example, review the literature on the effect of the microbiome–gut–brain axis on OCD onset.

Altogether, a strictly dimensional approach emphasizes the role of OCD as a crossroads of different pathogenic pathways. In developmental years, OCD compulsions could represent an evolutionarily conserved, “homeostatic” response over both endogenous and exogenous sources of unpredictability. This may have important clinical and therapeutic implications, as early OCS may mask underlying psychopathological vulnerabilities and interact with different developmental trajectories, shaping their course.
